# Genotype-by-genotype interactions reveal transcription patterns underlying resistance responses in Norway spruce to *Heterobasidion annosum* s.s

**DOI:** 10.1186/s12870-025-07438-1

**Published:** 2025-10-06

**Authors:** Hernan D. Capador-Barreto, Guus van Iersel, Mikael Brandström Durling, Jan Stenlid, Bo Karlsson, Malin Elfstrand

**Affiliations:** 1 Department of Forest Mycology and Plant Pathology, Swedish University of Agricultural Sciences, Uppsala, Sweden; 2 Smithsonian Tropical Research Institute, Balboa, Panama; 3 Microbiology, Department of Biology, Utrecht University, Utrecht, the Netherlands; 4 Skogforsk, Ekebo 2250, Svalöv, Sweden

**Keywords:** *Picea abies*, Quantitative disease resistance, Root rot, EamA-like transporter, UMAMIT family, NB-ARC, LRR

## Abstract

The expression of the genetic components involved in quantitative disease resistance (QDR) are often not well understood and analyses of genotype-by-genotype interactions can shed light on how the resistance and the activation of resistance strategies vary across interactions. To deepen the knowledge on QDR and the molecular interactions in the Norway spruce (*Picea abies*)- *Heterobasidion annosum s.l.*. pathosystem we inoculated ten relatively resistant spruce clones with four *H. annosum s.s.* isolates with varying virulence and determined the disease phenotypes. Thereafter we analysed the transcriptome in nine interactions between three spruce clones and three isolates. We tested the hypotheses that (i) differences among host and pathogen genotypes affect the disease symptoms; (ii) host genotypes respond differently depending on the isolate they were challenged with and (iii) this effect is accompanied by distinct transcriptional reprogramming. We found varying levels of disease expression among the interactions influenced by both host and pathogen genotypes. The host genotype was the strongest predictor of the disease phenotype, especially early in the interaction. The transcriptional responses differed between the three clones, interactions resulting in longer necrotic lesions were associated with larger numbers of differentially expressed genes. Despite this and the strong impact of the host genotype on disease phenotype each interaction displayed distinct transcriptional responses in a host genotype. These results indicate that phenotypic resistance responses can be governed by different defence modules and genes in Norway spruce. For instance, several leucine rich repeat family genes were found to be upregulated in the clones with higher resistance and downregulated in the clone with the lower resistance indicating that recognition of the pathogen may be one of the keys to limiting *H. annosum s.s.* spread in Norway spruce.

**Supplementary Information:**

The online version contains supplementary material available at 10.1186/s12870-025-07438-1.

## Introduction

In plant–pathogen interactions, disease symptoms arise from complex interactions between diverse molecular pathways in both host and pathogen in a conducive environment [1]. Therefore, the consequence of these interactions depends on the variation of pathways encoded in the host and pathogen genomes [2, 3]. Key processes in plant defence responses are recognition of an intruder, signal transduction and transcriptional reprogramming. In contrast to the well-characterized and well-described qualitative pathosystems, quantitative plant–pathogen interactions exhibit a lack of large-effect virulence/resistance genes that explain large proportions of the variance in the disease outcome in the population. Thus, quantitative interactions and disease resistance (QDR) typically present continuous distribution of phenotypes from susceptible to resistant [4–8]. The genetic variation underlying quantitative interactions reaches beyond host–pathogen perception and variation at large-effect loci which may create widespread differences in the metabolic and phenotypic responses to the interaction in the host and pathogen [3, 5, 7–9]. Quantitative interactions can be viewed as complex networks integrating multiple response pathways to several pathogen or host factors and environmental cues generating a continuous variation of phenotypes [1, 5, 6, 8, 10–12].

The host and pathogen genetic components involved in the bidirectional networks governing QDR are often not well understood and transcriptomic analyses of genotype-by-genotype interactions are useful to gain a broad picture of the components and regulatory complexity of the networks leading to disease or immunity in quantitative interactions [2, 13, 14]. Resistance to several of the major diseases in forest trees are having large components of QDR [15].

The conifer-*Heterobasidion annosum s*.*l.* are among the best studied forest pathosystems because they are of economic importance to the European forest industry [16]. The fungi cause root rot and stem decay primarily in Norway spruce (*Picea abies* (L. Karst) reducing growth, devaluating timber and making trees more susceptible to wind throw. The *H. annosum s*.*l.* species complex comprises five necrotrophic pathogenic decay fungi with different host ranges and distributions across the northern hemisphere [16, 17]. Three species, *H. annosum* s.s., *H. parviporum* and *H. abietinum*, are present within the natural distribution of Norway spruce (*Picea abies* (L. Karst)) and although *H. parviporum* is considered the primary pathogen on Norway spruce, *H. annosum* s.s., also readily infect this species [16, 17]. The genetic components of virulence on Scots pine (*Pinus sylvestris* L.) and Norway spruce have been characterized in members of the *H. annosum s*.*l.* species complex [18–20]. In *H. annosum s.s*. candidate genes related to toxins and xenobiotic catabolizing enzymes have been associated to virulence [20]. Analogously, the genetic components of resistance to *H. parviporum* and *H. annosum* s.s. have been characterized in Norway spruce [21–26]. This includes multiple QTL regions associated with successful defence to the pathogen including prevention of infection (Infection prevention, IP, where no viable fungus can be cultivated despite significant necroses in the phloem [23]), exclusion of the pathogen and control of the spread of the necrotic lesion (Lesion length, LL) in the bark and the pathogen in the sapwood (fungal sapwood growth, SWG) [23]. The QTL regions comprise for instance genes encoding enzymes producing antimicrobial compounds (e.g. PaLAR3 a leucoanthocyanidin reductase) or potentially cross-linking cell wall structures [21, 23, 27, 28]. The phenotypic and genotypic variation in interactions between Norway spruce and *H. annosum s*.*l.* is consistent with quantitative interactions, involving many genes with small to moderate effects in both partners. This is also supported by a study of the impact of genetic variation in Norway spruce and in *H. parviporum* on disease phenotype. Both the host genotype and the pathogen isolate significantly affected the size of the necrotic lesion in the bark [29].

Transcriptional analyses of Norway spruce in interaction with different members of the *H. annosum s.l* species complex have suggested broad transcriptional changes with similarities to the responses to wounding or infections with saprotrophic fungi, which changes as the infection progresses over time [30–35]. Distinct regulation of genes in specific host genotypes or tissues after challenge with members of the *H. annosum* s.l species complex have also been reported [21, 27, 36]. Collectively these observations show that Norway spruce recognizes *H. annosum* s.l and reprogram it transcriptome response. However, it is not known if Norway spruce regulates its transcriptome differently depending on the virulence of the pathogen, and whether this modulation is dependent on the host genotype and disease progression as it could be expected if QDR is a complex of networks integrating multiple response pathways generating a continuous variation of phenotypes [1, 5, 6, 8, 10–12].

The overall objective of this study was to investigate if variation in virulence in *H. annosum s.s.* would induce different responses in Norway spruce genotypes with relatively good resistance to natural infections by *Heterobasidion annosum s.l.*. To attempt to shed light on how the resistance and the activation of resistance strategies varied across host genotype -pathogen genotype interactions we hypothesised that (i) differences among host and pathogen genotypes affect the disease symptoms; (ii) host genotypes respond differently depending on the isolate they are challenged with and (iii) this effect is accompanied by distinct transcriptional reprogramming. To test these hypotheses we capitalized on one of the few genetic studies testing host resistance to naturally occurring *H. annosum* s.l [37] selecting ten clones showing higher resistance than the average population in the trial for this study. We generated grafted plants from the selected Norway spruce genotypes with varying levels of resistance to *H. annosum s.l.* [37] and inoculated them with five *H. annosum s.l.* isolates with varying levels of virulence [20] and analysed the transcriptome from the early and later stages of interactions between three host genotypes and three pathogen isolates.

## Materials and methods

### Plant material and fungal isolates

Grafts of ten Norway spruce clones in the South Swedish Norway spruce breeding program (0427, 1402, 1977, 2405, 4195, 4492, 4632, 4820, 4886 and 8590) with low to intermediate disease incidence of *H. annosum s.l.* in the clone trial S21S842979 [37], were generated and grown in Skogforsk’s experimental nursery at Ekebo, Sweden. In the nursery the grafted plants were subjected to standard watering and fertilisation. Five years after grafting, in June 2018, the plants were transferred to a greenhouse at SLU’s campus in Uppsala (20 ramets per genet). The plants were re-potted into standard cultivation soil in 33 cm diameter 15 L pots and randomized in the greenhouse. The greenhouse had a light regime of 16 h light and 8 h darkness at around 200 µmol in intensity. Temperature varied between 20 and 25 °C.

Four homokaryotic *H. annosum* s.s. isolates (L12-1, Rb28-20, Sä16 − 4 and 87087/8) with varying virulence in Norway spruce [20] were selected together with *H. parviporum* Rb175. Rb175 has been routinely used as the tester isolate in Norway spruce inoculation experiments [22, 38]. Isolate 87,087/8 was collected from a pine in Wettingen, Zürich, Switzerland by Ottmar Holdenrieder. The other three isolates were collected by Jan Stenlid, Rb28-20 was collected from a spruce in Ramsåsa, Sweden and Sä16 − 4 from a spruce in Sätuna, Sweden. Finally L12-1 was collected from a Sitka spruce (*P. sitchensis*) in Scotland, UK [20]. Each isolate was cultivated on Hagem’s media plates [39] for three weeks prior the experiment together with 5-mm sterilized Norway spruce wood plugs as previously described [40].

### Inoculations, lesion length determination and sample collection

**Fig. 1:**
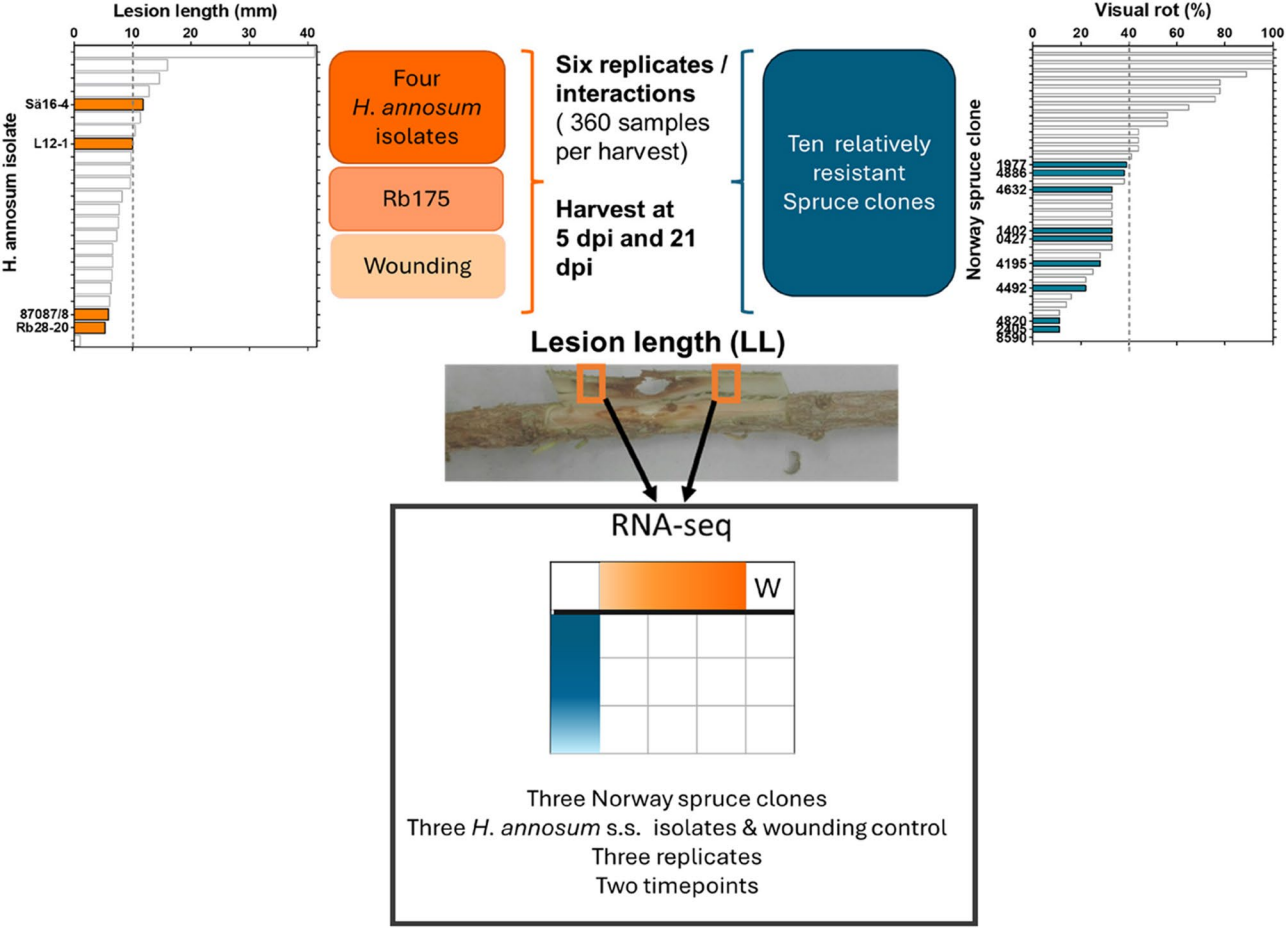
Experimental set up. four *H. annosum* s.s. isolates (orange bars) were selected based on the results of [20], ten Norway spruce clones (blue bars) with average or better resistance to natural infections (measured as the percentage of ramets with visual rot at stump height at 24 years of age) [37] were chosen for the experiment. Wounding control and inoculation with the *H. parviporum* Rb175 tester isolate were also included. Six replicates of each interaction were harvested at 5 and 21 dpi. At each harvest 5 × 5 mm bark samples adjacent to the necrosis were collected for transcriptome analysis

Artificial inoculations were performed on branch internodes from 2017 with the different isolates in an incomplete block design including a wounding control and two timepoints (Fig. 1). Every treatment (Isolate, or wounding) was repeated 6 times and present at least once together in the same ramet. At inoculation, bark was removed with a 6-mm diameter cork borer at the middle of the growth from 2017. A wooden plug colonized by *Heterobasidion* sp. was then placed at the wound and covered with Parafilm^®^ [29, 41] At harvest, the lesion length (LL) upwards and downwards from the edge of the inoculation point on the inside of the bark was measured at the second harvest the spread of the fungus in the sapwood (SWG) was also assessed. This was used to determine the proportion of failed inoculations, prevented (IP) and successful infections [23]. Likelihood-ratio tests (LTR test)were used to test the influence of host and pathogen genotype on lesion lengths at 5 and at 21 dpi. At each harvest, 5 × 5 mm squared bark samples were taken from both edges of the lesion and flash frozen in liquid nitrogen as described in [42]. The samples were stored at −80^◦^C until processing.

### RNA extraction and sequencing

We selected nine interactions at two time points for RNA sequencing based on their phenotype: 8590 (most resistant in the field, short LL in this study), clone 427 (moderately resistant in the field, short LL in this study) and 1977 (susceptible in the field, long LL in this study) in interactions with the isolates Sä16 − 4, 87,087/8 and L12-1. Wounding treatments of the clones were also harvested at 5 and 21 dpi to study expression induced specifically by *H. annosum s.s.* Total RNA was extracted as follows: samples were ground in liquid nitrogen with silica sand, and RNA was extracted according to [43] but without spermidine in the extraction buffer. The samples were treated with DNase I (Sigma-Aldrich) to eliminate contamination of genomic DNA. After checking for quality of the RNA with the Bioanalyzer, three biological replicates per condition, in total 72 samples, were sent for library preparation and subsequent RNA–sequencing at the Science for Life Laboratory in Uppsala, Sweden. Briefly, sequencing libraries were prepared from 300 ng total RNA using the TruSeq stranded mRNA library preparation kit (Cat# 20020595, Illumina Inc.) including polyA selection and sequenced in paired-end with 150 bp read length in a NovaSeq 6000 system, with a S4 flowcell and v1 sequencing chemistry.

### Quality control and mapping to the Norway spruce and *H. annosum s.s.* genomes

The quality of the reads was checked with FastQC, and quality reports were generated using MultiQC. Thereafter, raw reads were processed using trimmomatic v0.39 with ILLUMINACLIP: TruSeq3-PE.fa:2:30:10:8, TRUE LEADING:3, TRAILING:3, SLIDINGWINDOW:4:15, MINLEN:36 [44]. Trimmed reads were aligned to the *P. abies* genome v 1.0 gene models [45] using STAR default settings [46].

Reads were also aligned to the *H. annosum* s.s. genome (Durling et al. unpublished results), and gene counts were quantified using STAR (version 2.7.4a) with quantMode GeneCounts --alignIntronMax 5000 -alignMatesGapMax 5000 --alignIntronMax 5000 [46]. Since we are aware that samples contain wrongly mapped genes that probably belonged to either the host or other organisms in the tissue, we calculated the amount of “zero counts” per sample: the number of genes in the *H. annosum s.s.* genome with less than one read mapped them. Using this metric, we pruned samples having more than 75% genes with zero counts, (which included all wounded samples) for the subsequent analyses.

### Differential expression analysis

Unnormalized gene counts from STAR (version 2.7.4a) were used as input to perform differential gene expression analysis with the DESeq2 package in R (version 1.38) [47]. In DESeq2 genes with less than 10 total counts were filtered out of the dataset. Principal component analysis (PCA) of the samples was performed to explore the variation of the transcripts in the Norway spruce dataset.

Differential expression analysis in Norway spruce was performed for all factors in the experiment (Time, Host, Pathogen) using Wald tests or likelihood ratio tests (LRT) for significance testing (see below), and p values were adjusted for multiple testing using the Bonferroni procedure (*p* < 0.05). We used a small log2 fold change threshold of 0.5 in our analyses. For time, differential expression was calculated within timepoints between infected and wounded samples (genes consistently and differentially expressed in response to *H. annosum* s.s. in comparison to wounding, 5 dpi and 21 dpi). For host identity, an LRT was used where significance was defined as being differentially expressed in at least one host, and the same was done with pathogen identity.

### Network analysis

Variance stabilized gene counts were analysed in WGCNA [48] to build gene expression networks for both Norway spruce and *H. annosum s.s*. Based on the connectivity of the genes, a soft-threshold 10 was chosen to power the correlation of genes and build adjacency matrices. The minimum module size was set to 30, a similarity threshold of 75% was used to merge modules together, and the default unsigned option was utilized. Gene modules in the host were correlated with gene in modules in the pathogen to identify genes in Norway spruce that are correlated with changes in expression in *H. annosum s.s*. To avoid the correlation of modules with genes belonging to *P. abies*, but wrongly mapped to the *H. annosum s.s*. genome we removed modules in the pathogen which were correlated to the number of zero counts.

## Results

### Lesion development in the inner bark depends on Norway spruce and *H. annosum* genotypes

**Fig. 2:**
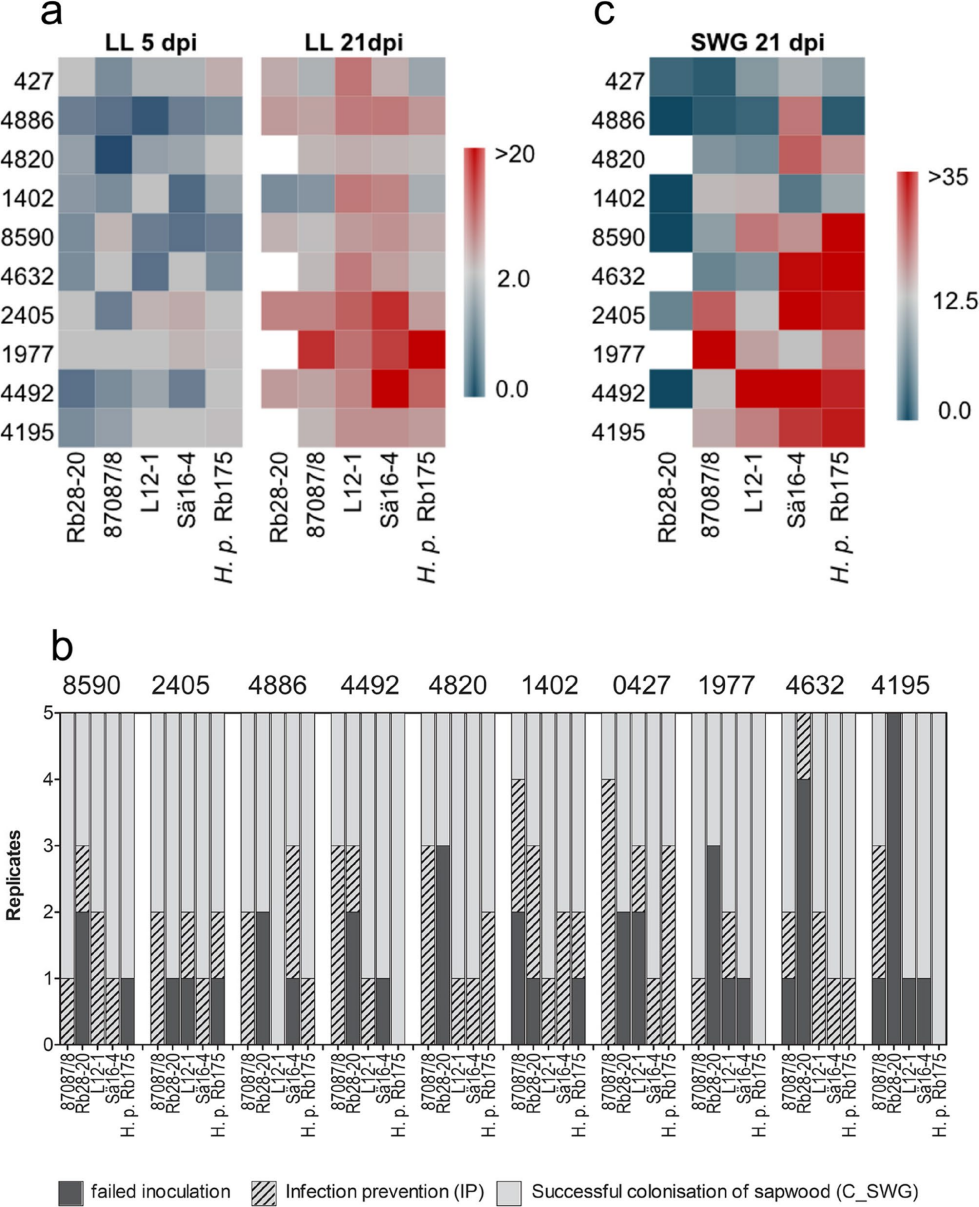
Results from the inoculation experiment. The heatmaps in (**a**) show the mean lesion length (LL) in mm at 5 and 21 dpi for each host-pathogen combination. The histograms in (**b**) show the number of failed inoculations (dark grey), prevented infections (hashed bars) and inoculations with colonization of the sapwood (light grey) at 21 dpi. The total number of inoculations per host-pathogen combination was five. The heatmap in (**c**) show the mean fungal spread in sapwood (SWG) in mm at 21 dpi for each host-pathogen combination

The mean lesion length was 1.7 mm (+/- 1.9 mm) at 5 dpi following inoculation of Norway spruce with *H. annosum s.l.*, and at 21 dpi the mean lesion had grown to 5.4 mm (+/- 6.3 mm). Three of the Norway spruce clones (1977, 2405 and 4886) allowed longer lesions at 21 dpi compared to at five dpi, while host 0427 allowed almost no progression of the lesion length between the first and second timepoint (Fig. 2). At both timepoints the *H. annosum* s.s. isolate Rb28-20 produced shorter mean lesions than L12-1 and Sä16 − 4 (Fig. 2). At the first time point (5 dpi) the host genotype had a significant effect on the observed variation in lesion length (LRT tests, Table 1). At the second time point, 21 dpi inoculation, both the host and the pathogen genotypes showed significant effects on the observed lesion length (Table 1). However, the interaction term was not significant (*p* = 0.13, Table 1). Mean values per combination at both timepoints showed a weak but significant correlation (*R* = 0.31, *p* = 0.02), suggesting that the disease outcome measured as progression of the lesion length was generally shaped early in the interaction.

**Table 1:** Likelihood-ratio test (LTR test) statistics of the lesion lengths in the inner bark of Norway Spruce at 5 and at 21 Dpi to determine which factors that influence the phenotypes significantly

Treatment	LRT statistic	Df	*P*-value
5 dpi			
Host	17.55	9	0.04*
Pathogen	3.98	4	0.40
Host x Pathogen	25.78	36	0.89
21 dpi			
Host	47.341	9	3.39e-07 ***
Pathogen	34.524	4	5.82e-07***
Host x Pathogen	45.575	36	0.13

At 21 dpi the spread of the fungal isolates in the sapwood was also recorded and this allowed the assessment of the proportion of failed inoculations, prevented infections and successful infections. This analysis indicated that the two less virulent isolates Rb28-20 and 87,087/8 established successful interactions in only 40% and 50% of the inoculations, respectively. In fact, the isolate 87,087/8 was prevented from reaching the sapwood in almost 42% of the inoculations that were made (Fig. 2). For the more virulent *H. annosum* isolates and the *H. parviporum* isolate approximately 70% of the inoculations reached the sapwood successfully. There were no significant differences in the fungal progression in the sapwood at 21 dpi (Fig. 2).

### Norway spruce transcriptional responses are mainly dependent on host genotype, time after infection, and lesion length

For the transcriptome analysis we selected the interactions between three Norway spruce clones and three *H. annosum* isolates and the wounding controls. One Norway spruce genotype had very high reported field resistance but with limited capacity to control lesion length development and spread in the sapwood in inoculation tests (8590) (Fig. 2a and b). The two other two Norway spruce genotypes had lower reported field resistance and showed marked differences in lesion length development and spread in the sapwood: The genotype 0427 displayed higher resistance in the inoculation tests and 1977 was markedly more susceptible than the other two clones in the inoculation study (Fig. 2). We sampled green healthy looking phloem tissue adjacent to the necrosis margin from these three clones challenged with three *H. annosum s.s.* isolates (Fig. 1), and wounding alone was sampled as a control. Two of the isolates that we used are highly virulent (Sä16 − 4 and L12-1) and the third, 87,087/8, less virulent according to [20] and our inoculation study. Sequencing generated on average 38.53 M reads per sample after quality filtering. On average, 76.4% of these reads mapped to the Norway spruce genome (Supplementary Table 1). A principal component analysis (PCA) of the counts per Norway spruce gene and sample indicated that samples were clustered based to the Norway spruce genotype and the time after infection (Supplementary Fig. 1). PC1, which explained 51% of variation showed a moderately strong correlation to the lesion length (*R* = 0.55, p value = 7.76e-07, Supplementary Fig. 1). Total read counts mapped to the *H. annosum s.s* genome ranged from 755 reads to 1.08 M reads per sample or on average 0.49% of the reads. Based on low coverage, samples with insufficient fungal RNA were filtered out (Supplementary Fig. 2). This left 17 samples of the original 72 with detectable *H. annosum s.s* transcripts (Supplementary Fig. 2). Interestingly there was a trend that the more virulent isolates more frequently had detectable expression when compared to the least virulent isolate 87,087/8 (Chi-square = 5.323, df = 2 *P* = 0.07). This is consistent with that this isolate less frequently established successful interactions (Fig. 2b). The fungal gene expression patterns showed some separation between pathogen genotypes and times but not host genotype (Supplementary Fig. 2).

**Fig. 3:**
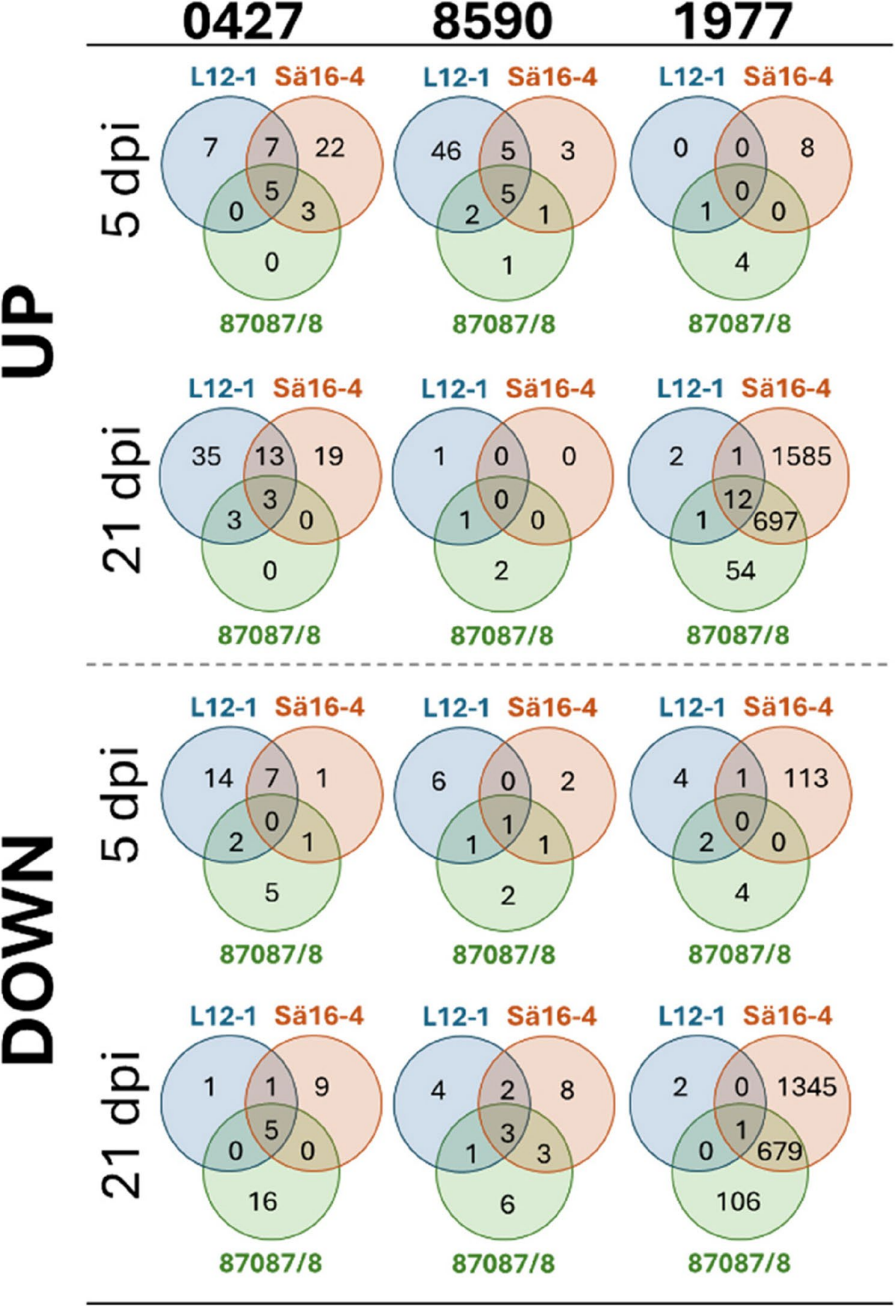
Up and down regulated differentially expressed genes (DEGs) in the nine interactions in the transcriptome study. Each column of Venn diagrams represents a clone (0427, 8590 or 1977). The two rows of Venn diagrams above the hashed line present upregulated DEGs at 5 dpi and 21 dpi respectively. Analogously the two rows of Venn diagrams below the hashed line present downregulated DEGs at 5 dpi and 21 dpi respectively. The blue circles in the Venn diagrams are the DEGs in the clones’ interaction with *H. annosum* s.s. L12-1, the orange circle represent interactions with *H. annosum* s.s. Sä16 − 4 and green with *H. annosum* s.s. 87,087/8

### Transcriptional responses in the clones are different and often isolate specific

In total 11,495 Norway spruce genes where differentially expressed in at least one host genotype, this corresponds to 22.5% of the annotated gene models. Several genes previously shown to be part of Norway spruce induced defence responses were found to be upregulated in response to inoculation compared to wounding in the bark when all genotypes and isolates were combined. For instance, *PaLAR1* (MA_7866760g0010), *ACC synthase* (MA_121240g0010) and *PaMYB29* (MA_9818613g0010) were upregulated at 5 dpi and several *class IV chitinase* genes at 21 dpi (Supplementary Table 2). When every host - pathogen combination was compared against the relevant wounding control at the two timepoints (Fig. 3, Supplementary Table 3) a more complex picture emerged. None of the significantly differentially expressed genes compared to the wounding control were found to be differentially expressed in every host-pathogen combination (Supplementary Table 2, Supplementary Table 3). A threshold of +/- 20 log2 fold change was applied to the DEG in each interaction leaving in total 47 strongly up- or downregulated DEG in at least one interaction underlining the clone-based differences (Table 2).To identify the broad patterns in each interaction, PFAM enrichment analyses were done for up and down regulated DEGs in each interaction (Supplementary Table 4).

### The clones 8590 and 0427 had different trajectories of disease development yet their transcriptional responses showed similarities

The interactions between the clones 0427 and 8590 and the least virulent isolate 87,087/8 were consistent with the host being able to control the infection. At 21 dpi, the fungus had generally not reached the sapwood in 0427, and in 8590 the colonization of sapwood was limited (Fig. 2c). The interaction between the clones 8590 and 0427 and the isolate 87,087/8 were also the only interactions where the measured lesion had not progressed at 21 dpi compared to 5 dpi at (Fig. 2a and c). This was also reflected in limited transcriptional changes in both these interactions (Fig. 3, Supplementary Table 2). Infection with the two more virulent *H. annosum* isolates (Sä 16 − 4 and L12-1) on 8590 and 0427 was accompanied by larger, but still modest, transcriptional changes at five and 21 dpi respectively (Fig. 3, Supplementary Table 3). There were generally more upregulated genes early in these interactions than in interactions with the less resistant genotype 1977 and the upregulated genes also appeared more strongly upregulated at 5 dpi in 8590 and 0427 (Table 2 and Supplementary Table 3). MA_102298g0010 (peroxidase 66) presented an exception its induction levels in interactions were equally strong in all interactions with 87,087/8 at 5dpi (Table 2, Supplementary Table 3). Among the strongly differentially expressed genes, some showed distinct expression patterns, MA_10370048g0010 (*1-aminocyclopropane-1-carboxylate oxidase*), MA_10374232g0010 (*homeobox-leucine zipper ROC3-like isoform X1*) and MA_58978g0010 (Lipid transfer protein), were upregulated and MA_90135g0010 (encoding a polygalacturonase-like protein) downregulated specifically in 8590 at 5dpi (Supplementary Table 3). Similarly MA_10433639g0010 were specifically upregulated in 0427 at 5 dpi (Table 2)These patterns may suggests that the genes are part of defence modules used differently in the specific clone than in in the other two clones.

**Table 2:**
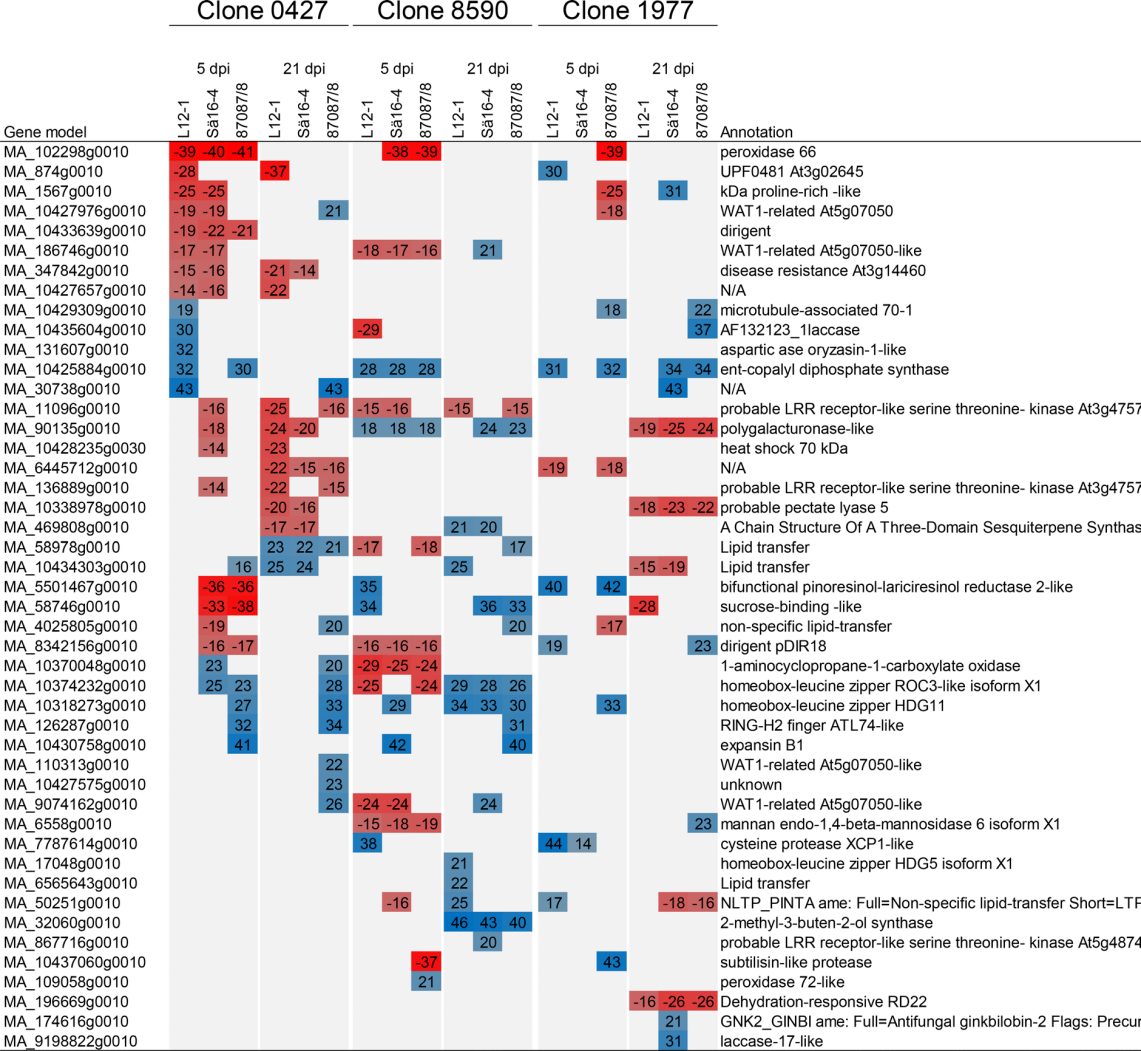
Strongly differentially expressed genes (> 20 and < −20 log2foldchange) compared to wounding at 5 and 21 Dpi (days post inoculation) in every host and pathogen combinations

The PFAM domains PF13855 (Leucine rich repeat), PF13306 (Leucine rich repeats (6 copies)) and PF00560 (Leucine Rich Repeat) were enriched among upregulated genes in interactions at 21 dpi in clones 0427 and 8590 (Supplementary Table 4). Furthermore, MA_11096g0010, an leucine rich repeat encoding protein gene (LRR) and probable LRR-XII receptor-like kinase [49] was strongly upregulated in 0427 and 8590 in several interactions at both timepoints, but never in 1977 (Table 2, Supplementary Table 3). In clone 0427 MA_347842g0010, also with similarity to a NB-LRR family disease resistance gene (At3g14460), was strongly upregulated in interactions at both timepoint the gene was not significantly regulated in the other clones (Table 2, Supplementary Table 3). The interaction between 0427 and L12-1, which generated relatively long lesions at 21 dpi, both in comparison to the lesions at 5 dpi and in other interactions with the clone (Fig. 2) the number of DEGs, mainly upregulated DEGs, increased from 5 to 21 dpi. The upregulated DEGs were also enriched for PFAM domains associated to terpenoid biosynthesis (PF01397 and PF03936) and receptor like kinases (PF13306, PF13855 and PF00069) (Supplementary Tables 3 and 4).

MA_186746g0010 (*WAT1-related At5g07050-like*) and MA_8342156g0010 (dirigent *pDIR18*) were upregulated in at least two interactions in 0427 and 8590 (Table 3). The PFAM enrichment analyses showed relatively consistent enrichment patterns in interactions with the three pathogen isolates, especially at 5 dpi (Supplementary Table 4). For instance, PF3018 (dirigent proteins) and PF0089 (EamA-like transporter family) were enriched among upregulated DEGs, and PF03936 (Terpene synthase family, metal binding domain) among downregulated DEGs in most interactions involving 0427 and 8590.

### The more susceptible clone responds differently and the transcriptional response change with the lesion length

In the more susceptible clone, 1977, all isolates generated long lesions at 21 dpi, in particular Sä16 − 4 and 87,087/8 (Fig. 2a). These isolates were also successful in colonizing the sapwood at 21 dpi (Fig. 2c). The interaction between 1977 and Sä16 − 4 also showed the largest number of DEGs at 5 dpi, all down regulated compared to the wounding control (Fig. 3). Consistent with the disease phenotype, Sä16 − 4 and 87,087/8 inoculation led to large transcriptional changes in 1977 at 21 dpi (Fig. 3, Supplementary Table 3). Most of the genes that were differentially expressed in response to isolate 87,087/8 (89%) were also differentially expressed in response to the more virulent isolate Sä16 − 4 at this timepoint (Fig. 3, Supplementary Table 3). The vast majority of these DEGs showed small to moderate up and downregulation, but 31 and 36 DEGs were strongly differentially regulated at 21 dpi in the interaction of 1977 with 87,087/8 and Sä16 − 4 respectively (Table 2, Supplementary Table 3).

The PFAM enrichment analysis (Supplementary Table 4) showed that in interactions between 1977 and 87,087/8 or Sä16 − 4 at 21 dpi, domains associated with plant defence responses were enriched among the upregulated DEGs with both isolates. Examples include PF00182 (Chitinase class I) PF00255, (Glutathione peroxidase), PF00332 (Glycosyl hydrolases family 17) and PF00314 (Thaumatin family) (Supplementary Table 4). The downregulated DEGs in interactions between 1977 and both 87,087/8 and Sä16 − 4, were enriched for PF13855 (Leucine rich repeat) and several other PFAM domains associated with receptor like kinases (PF00931-NB-ARC domain, PF00560-Leucine Rich Repeat, PF08263-Leucine rich repeat N-terminal domain and PF13516-Leucine Rich repeat) (Supplementary Table 4).

### Gene networks in the host are correlated with gene networks in the pathogen

**Fig. 4:**
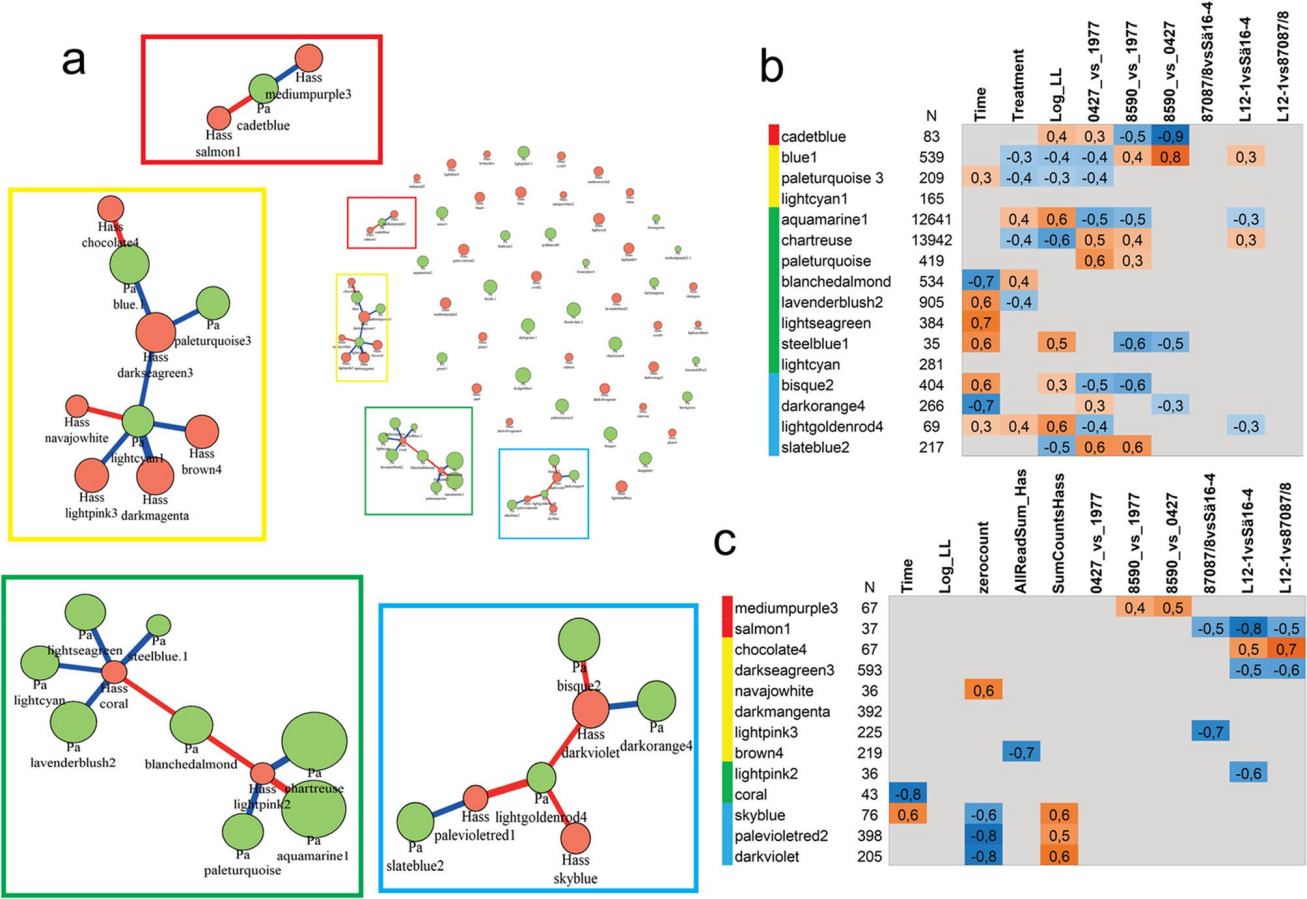
Correlation of Norway spruce gene expression modules (green nodes) and *H. annosum *gene expression modules (orange nodes) (**a**). The size of the nodes reflects the size of the modules and the thickness of the edges the strength of the correlation between modules. Red edges indicate positive correlations and blue edges negative correlations. Four co-expression networks (red, yellow, green and blue are indicated. The module-trait correlations for the Norway spruce modules in the red, yellow, green and blue networks are shown (**b**), positive and significant module-trait correlations are indicated in orange colours and negative significant correlations in blue, grey indicate lack of significant correlations. Similarly, the *H. annosum* module-trait correlation in the four co-expression networks are shown in (**c**) positive and significant module-trait correlations are indicated in orange colours and negative significant correlations in blue, grey indicate lack of significant correlations

Despite the very limited number of detected *H. annosum* s.s. transcripts we attempted to build two expression networks with genes mapping to the host or pathogen genome. In the host, genes were grouped into 36 modules with varying size (48 to 13942 genes per module). In the pathogen we initially identified 21 modules. We correlated the gene modules in the host to gene modules in the pathogen to identify network components in the Norway spruce interacting specifically with *H. annosum s.s* (Fig. 4). Most of the fungal and host modules did not correlate, but four small correlation networks were found, called yellow, green, blue and red (Fig. 4). It is worth noting that the *H. annosum* s.s. modules in the blue network appear to be governed by the read depth (Fig. 4a and c).

An analysis of the relation between the modules in these networks and the differentially expressed genes showed that the majority of the DEGs were found in modules in the yellow and green network (Fig. 4, Supplementary Table 5). In the green network the two small fungal modules Hass coral and lightpink2, each comprising less than 50 fungal transcripts, interact with several relatively large host modules. The modules “Pa aquamarine 1” and “Pa chartreuse” correlate positively respective negatively with the fungal module “Hass lightpink 2”(Fig. 4a). An inspection of the module trait-relationships and expression patterns in the modules indicated that these interactions may bedriven by the interactions between the less resistant host 1977 and primarily *H. annosum* isolate Sä16 − 4 (Fig. 4b and c, Supplementary Table 5)which also showed the largest transcriptional profile changes.

In the yellow network, three host modules “Pa blue 1”, “Pa paleturquoise 3” and “Pa lightcyan 1” correlates negatively with the fungal module “Hass darkseagreen3” which is characterized by negative correlations with L12-1 inoculation (Fig. 4a and c). The module “Pa Blue 1”, also show positive correlation to the fungal module “Hass chocolate4”, characterized by positive correlations with the L12-1 inoculations. (Fig. 4a and c), perhaps not surprising as L12-1 inoculations represented almost 50% of the samples with *H. annosum* reads (Supplementary Table 2) used in the expression network. The module “Pa blue 1” in the yellow network comprised 539 expressed genes (Fig. 4b, Supplementary Table 5). The module includes a number of LRR- or receptor-like protein genes with a strong modular belonging, for instance MA_10437181g0020 (7.7 e-14), MA_608524g0010 (1.8 e-18), MA_10432522g0010 (6.8 e-14) and MA_3635554g0010 (6.5 e-13) (Supplementary Table 5). The tlatter DEGs are also downregulated at 21 dpi in the interaction between 1977 and or *H. annosum* isolates Sä16 − 4 and 87,087/8 (Supplementary Table 3). In addition to the observation of several LRRs among the genes with strong modular belonging the “Pa blue 1” module is enriched for the PF00931-NB-ARC domain together with related domains such as PF13855-Leucine rich repeat and PF13306-Leucine rich repeats (6 copies) among others (Supplementary Table 6). Thus, there appears to be a potential enrichment of genes associated with immune signalling in the module. Interestingly “Pa paleturquoise3” also show sign of enrichment for PFAM domains associated with immune responses e.g. PF01764 and PF15699 (Supplementary Table 5). Strong modular belonging of genes associated with these domains can be observed e.g. MA_10437224g0020 (*EDS-like*; 0.039) and MA_7617132g0010 (*SUPPRESSOR OF npr1- CONSTITUTIVE 1-like* 0.011) (Supplementary Tables 5 and 6). MA_7617132g0010 and putative paralogs are also more highly expressed in the wounding control than in 1977 challenged by Sä16 − 4 (Supplementary Table 3).

## Discussion

The objective of this study was to understand how genetic -and phenotypic- variation in both partners drive expression of disease symptoms in the bark, hypothesising that both intraspecific variation in Norway spruce and *H. annosum* s.l. would contribute to variation in lesion length. The phenotyping results suggested that variation in resistance to *H. annosum* s.l. in the host was the main driver of the lesion length extension at both time points. The lesion length data also indicated that disease outcome was defined early in the interaction and that different specific interactions had different trajectories that determined e.g. successful colonization of the sapwood at 21 dpi. As the interaction progressed the genotypic variation in the pathogen also became evident and variation in mean lesion sizes among the isolates was detected. However, no interaction effect was observed at either time point. The result that host and pathogen genotypes, but not the interaction between them, significantly influenced the lesion length in *H. annosum* s.l. inoculated Norway spruce branches agrees both with previous work on interactions between Norway spruce and *H. parviporum* and with the general trend among genotype-by-genotype matrix analyses in plant-pathogen interactions; an additive effect on the resistance in the host and the pathogen and little or no interaction effect is common [2, 26, 29]. Albeit it is possible that our study included too few host-pathogen combinations or replicates to detect significant interactions between the host and pathogen genotype, our results enforces the conclusions from Swedjemark and Stenlid [29] that one or a low number of fungal isolates are sufficient in resistance testings. It is known that expression of QDR can vary between environments and with assay methods [50–52], and this study was carried out under controlled conditions in the greenhouse, conditions in the forest would be considerably more variable, including availability of water [28]. Therefore, it is encouraging that there was a weak positive correlation with the symptom severity in this study and previous field assessments [37].

Analyses of gene expression patterns in tree-pathogen interactions are often done aggregated over genotypes with the aim to detect general patterns [31, 34, 42, 53–55]. An analysis of differentially expressed genes ignoring the nine specific host- and pathogen genotype combinations in comparison to wounding revealed responses similar to previous work on interactions between Norway spruce and *H. annosum* s.l. Upregulation of known key genes *PaLAR1*,* PaMYB29*,* ACC synthase* and *class IV chitinases* in Norway spruce defence responses could be detected [30, 36, 56]. However, when analysing the expression patterns in the nine host-pathogen interactions separately they showed distinct transcriptional responses, not necessarily involving these previously identified key genes.

The observation that the variation in resistance in the host was the main driver of the lesion length extension is reflected in the transcriptional responses where the two clones that allowed relatively short lesions after 21 days of interactions showed more, mostly clone specific, upregulated genes at five dpi than the less resistant clone. Several of the upregulated DEGs in 0427 and 8590 were induced in interaction with two or more pathogen isolates. The less resistant clone did not display a similar core response of upregulated DEG. Only a single downregulated DEG was shared between two interactions at 5dpi in 1977.

Several of the strongly upregulated DEGs at 5 dpi in 0427 and 8590 showed similarity to WAT1 (walls are thin 1), including MA_186746g0010, and the corresponding PFAM domain (PF00892 EamA-like transporter/UMAMIT family) amino acid transporters was enriched among the upregulated genes at the first time point in these more resistant clones. Angiosperm members of this gene family have been identified as susceptibility factors for several vascular wilt pathogens and mutants are often impaired in plant hormone signalling and cell wall formation [57–60]. However, their role in resistance may be dependent on the interaction as mutations in a gene family member (*RTP1*) affect susceptibility to biotrophic pathogens but does not affect resistance to *Botrytis cinerea* [57]. MA_186746g0010 and a predicted dirigent protein (MA_8342156g0010-) which may be involved in secondary cell wall formation [61] were upregulated in five out of six interactions involving 0427 and 8590. The formation of a lignified and suberized boundary zone at is pivotal in defence responses in the bark [62], dirigent proteins and redox enzymes such as peroxidases are key players in the lignification process. The observed regulation of MA_8342156g0010 together with the observation that the gene model MA_102298g0010 encoding a peroxidase was strongly upregulated in all interactions in 0427 and 8590 at 5 dpi, makes it tempting to speculate that these clones enforce cell walls to limit the spread of the pathogen early in the interaction. MA_102298g0010 encodes a plant peroxidase which is highly similar to *PaPX17*, a peroxidase bound to the released lignin in lignin-forming Norway spruce suspension cultures [63]. At 21 dpi the pattern was reversed. The clone 1977 showing the longest lesions, showed very large numbers of, partly overlapping, up and downregulated DEGs in response to *H. annosum* s.s. Sä16 − 4 and 87,087/8 at 21 dpi consistent with broad, and possibly less effective, transcriptional reprogramming in response to the advancing lesion. This pattern that most of the host- and pathogen genotype combinations showed distinct transcriptional responses could be consistent with observations in other systems where similar host disease phenotypes can be governed by different defence modules and the disease outcome is determined early in the interaction [64, 65].

The clone 0427 displayed the resistance trait infection prevention, IP [23], more frequently than the other two clones. Interestingly we observed that several Leucine Rich Repeat (LRR) containing genes were strongly upregulated in multiple interactions and at both timepoints in this clone. Some of these differentially regulated LRRs appear to encode genes that belong to LRR-XII receptor like kinases [49]. MA_11096g0010 showed a similar pattern of regulation in 8590. In Arabidopsis the LRR-XII RLKs clade of LRRs includes well-known pattern-recognition receptors (PRRs) including FLS2 and EFR [49, 66, 67], indicating that these gene models may encode PRRs or receptors that recognize other molecules associated with the infection, even if one always should be cautious to make predictions based on similarity alone [68]. No gene models with similarity to LRRs showed similar patterns of strong upregulation in 1977, instead they were mostly downregulated at 21 dpi. Members of the *H. annosum* s.l. species complex are reported to both degrade the host cell wall with carbohydrate active enzymes and produce toxic metabolites and effector-like proteins that can induce cell death [69, 70]. It is possible that the differences in LRR expression between 0427 and 1977 reflects different capacities to detect and respond to the imminent threat of an inoculation or infection. As reviewed by Delplace and co-authors [8] recognition of an attack is crucial to QDR, and number of loci linked to QDR in other pathosystems are associated with perception of the pathogen as well as signalling [1, 71]. The success of the isolate 87,087/8 that has a relatively low virulence [20] on this clone and the small number of differentially expressed genes at 5 dpi compared to at 21 dpi also support that the activation of key defence modules is impacted in 1977 early in the interaction.

The virulence of the four *H. annosum* s.s. isolates used in this study on Norway spruce were previously determined in inoculation experiments [20]. It is established that different QTLs govern the virulence of *H. annosum* s.l. on Scots pine and Norway spruce, and that variation in virulence may be associated with copy number variations in the pathogen genome [19, 20, 72]. Two of the isolates displayed similar levels of virulence on Norway spruce in the current study while the other two deviated somewhat from the predicted phenotype [20]. It is possible that such quantitative variation is expressed differently between environments or hosts, despite the lack of statistical support for genetic interactions in this study. Interestingly it has been shown that in interactions between *B. cinerea* and Arabidopsis genetic variation in the pathogen has a greater impact on the co-transcriptome than mutated defence signalling pathways in the plant [14], pointing to the importance understanding also the molecular interactions in QDR. Unfortunately, in this study a very small fraction of the reads mapped to the *H. annosum* s.s. genome making detailed analyses of the fungal transcriptional responses to the confrontation with different host genotypes difficult. Dual RNA-seq analysis of interactions between conifers and *H. annosum* s.l. often generate very few reads from the pathogen, despite obvious necrosis in the tissue, making in depth analyses of the interaction transcriptome difficult [32, 55, 70, 73]. This may be a consequence of the sampling strategy in this, and many of the previous studies, where samples are taken adjacent to the necrotic lesion. *Heterobasidion annosum* s.l. biomass is often concentrated inside the necrotic lesion or the sapwood [74, 75] where the fungus can live saprophytically. It is however interesting that the samples with detectable *H. annosum* s.s. reads were dominated by the more virulent genotypes and that the gene expression patterns showed some separation between pathogen genotypes and times but not host genotype. Indicating that the there was a relationship between detectable *H. annosum* s.s. expression and the pathogens virulence and but less apparent interactions with the host genotype. Using the limited number of detected *H. annosum* s.s. transcripts we made a WGCNA to probe the expression modules in the two partners. Reflecting the limitations of the analysis only four very small networks were detected, and these appeared to be mostly driven by the large-scale transcriptional changes in the less resistant clone 1977 on the host side on the pathogen side the networks appeard to be driven by the read depth and the expression in L12-1. However, in this analysis the potential role of immune signalling and LRRs in interactions between Norway spruce and *H. annosum* s.s. was highlighted in one of the networks, the modules enriched for components of immune signalling generally showed significant relationships with the traits we measured in the study and differential regulation between interactions of key genes in the modules, indicating a potential role of these receptors and immune signalling in the observed phenotypic differences. Consequently, it would be interesting to follow up on the possibilities that variation in specific receptors or defence modules determine the level of quantitative disease resistance already early in the interaction between Norway spruce and *H. annosum s.s* in the progeny from controlled crosses between the three clones in this study, in particular in crosses between 0427 and 1977.

In conclusion, in this study we showed that different *H. annosum s.s.* isolates with varying levels of virulence induced different responses also in Norway spruce genotypes with relatively good resistance to *H. annosum s.l.*. We found varying levels of disease expression among the interactions influenced by host and pathogen genotypes, but not the interaction between them. This underlies the conclusions from Swedjemark and Stenlid [29] that a single fungal isolate in resistance tests is sufficient to capture the variation in resistance in the host. This picture shifted when analysing the transcriptional responses. The nine host- and pathogen genotype combinations showed distinct transcriptional responses. Not only did the transcriptional responses differ between the clone with the shortest lesions (0427) and the clone with the longest (1977) as could be expected under the assumption of variation in QDR. The response also differed between interactions with the three isolates within a clone despite relatively similar phenotypic responses. These results are consistent with the disease outcome being shaped early in the interaction and suggest that phenotypically similar host responses can be governed by different defence modules. This variability can to some degree explain the sometimes conflicting reports on key domains in host defence to *H. annosum* s.l. in literature and call for systematic studies on the early stages of the interactions and their associated phenotypic and molecular responses.

## Supplementary Information

Supplementary Material 1.

Supplementary Material 2.

Supplementary Material 3.

Supplementary Material 4.

Supplementary Material 5.

Supplementary Material 6.

Supplementary Material 7.

Supplementary Material 8.

## Data Availability

The raw sequence data are available in the BioProject PRJNA1253411.
