# Supplementary figures and images for: Genotype-by-genotype interactions reveal transcription patterns underlying resistance responses in Norway spruce to Heterobasidion annosum s.s
Source: BMC Plant Biol. 2025 Oct 6;25:1326. doi: 10.1186/s12870-025-07438-1 (PMC12502169; doi:10.1186/s12870-025-07438-1)

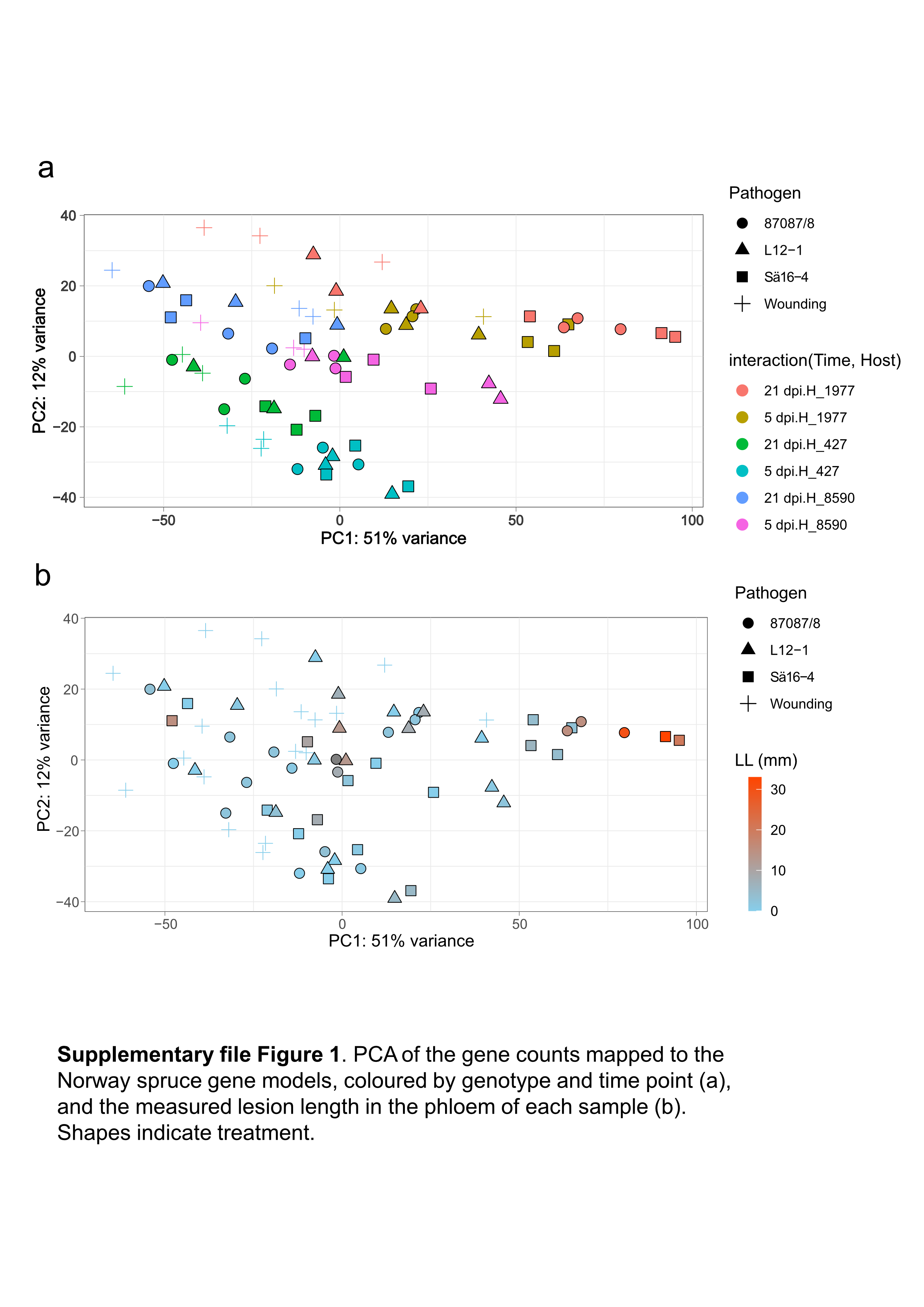

Supplement: Supplementary file 7 — Supplementary Material 7. [file 12870_2025_7438_MOESM7_ESM.tiff]

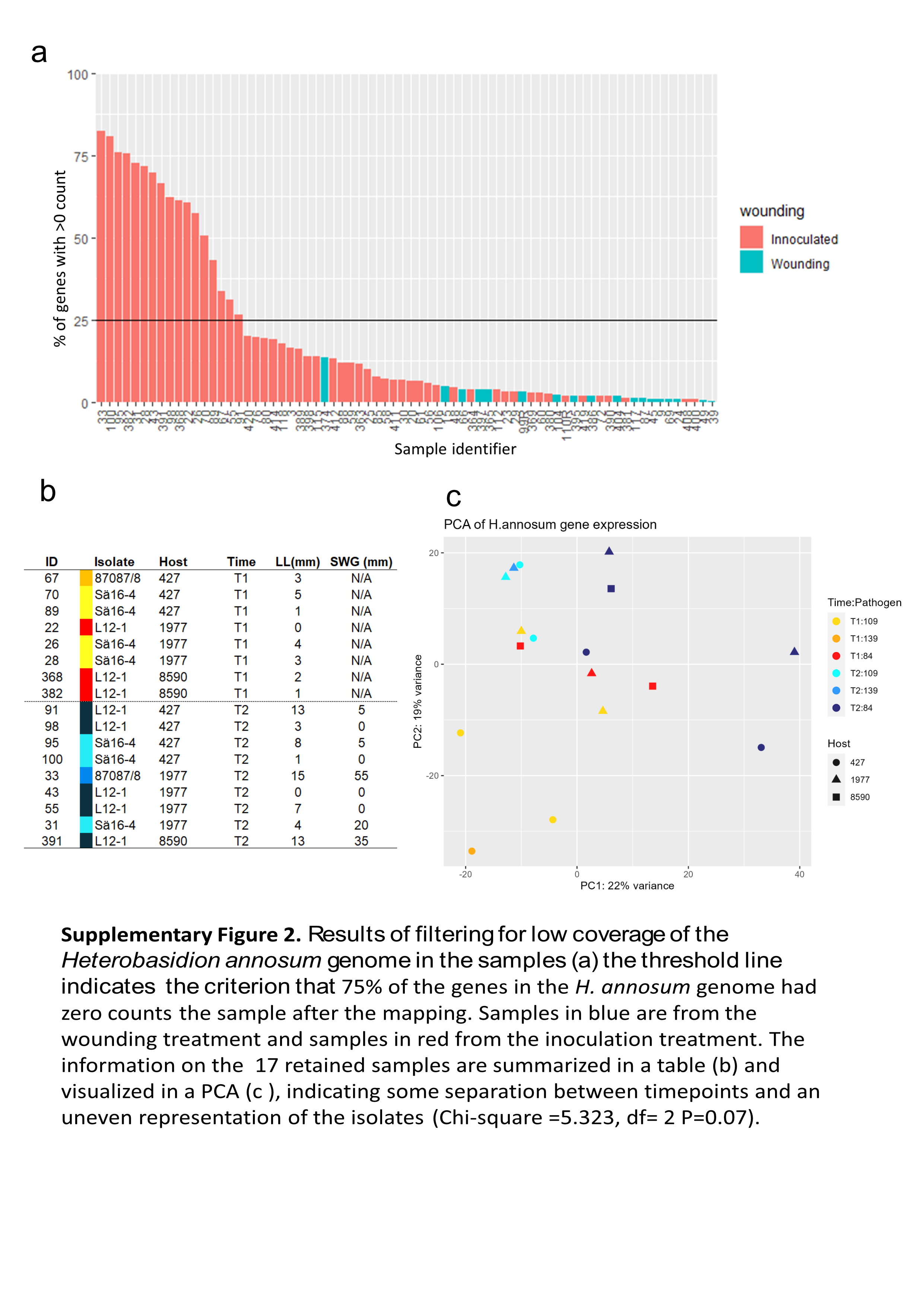

Supplement: Supplementary file 8 — Supplementary Material 8. [file 12870_2025_7438_MOESM8_ESM.tiff]
